# A systematic review of factors influencing NHS health check uptake: invitation methods, patient characteristics, and the impact of interventions

**DOI:** 10.1186/s12889-019-7889-4

**Published:** 2020-01-21

**Authors:** Amanda Bunten, Lucy Porter, Natalie Gold, Vanessa Bogle

**Affiliations:** 1 Public Health England, PHE Behavioural Insights Team (PHEBI), Research, Translation & Innovation Division, 6th Floor, Wellington House, 133-155 Waterloo Road, London, SE1 8UG UK; 2 University of Exeter, Exeter, UK; 3 City University, London, UK

**Keywords:** NHS health check, Cardiovascular disease, Primary prevention, Uptake, Attendance, General practice, Invitation

## Abstract

**Background:**

The NHS Health Check (NHSHC) is a risk assessment for those aged 40–74 without a pre-existing condition in England, with the aim of preventing stroke, kidney disease, heart disease, type 2 diabetes and dementia. Uptake has been lower than anticipated. Ensuring that a high percentage of eligible patients receive a NHSHC is key to optimising the clinical and cost effectiveness of the programme. The aim of this systematic review is to highlight interventions and invitation methods that increase the uptake of NHSHCs, and to identify whether the effectiveness of these interact with broader patient and contextual factors.

**Method:**

A systematic review was conducted according to the PRISMA checklist. Papers were eligible if they explored the impact of at least one of (i) interventions, (ii) invitation methods or (iii) broader factors on NHSHC uptake. Ten databases were searched in January 2016 and seven were searched in March 2018. Nine-hundred-and-forty-five papers were identified, 238 were screened and 64 full texts were assessed for eligibility. Nine studies were included in the review.

**Results:**

The nine studies were all from peer reviewed journals. They included two randomised controlled trials, one observational cohort and six cross-sectional studies. Different invitation methods may be more effective for different groups of patients based on their ethnicity and gender. One intervention to enhance invitation letters effectively increased uptake but another did not. In addition, individual patient characteristics (such as age, gender, ethnicity and risk level) were found to influence uptake. This review also finds that uptake varies significantly by GP practice, which could be due either to unidentified practice-level factors or deprivation.

**Conclusions:**

Further research is needed to assess the effectiveness of different invitation methods for different population groups. Research should examine how existing invitation methods can be enhanced to drive uptake whilst reducing health inequalities.

**Trial registration:**

This systematic review was registered with PROSPERO on 22.02.2016. Registration number CRD42016035626.

## Background

Cardiovascular disease (CVD) is the number one cause of death globally; an estimated 17.9 million people died from CVD in 2016, representing 31% of all global deaths [1]. It is estimated that 50 to 80% of CVD cases are caused by modifiable risk factors such as smoking, obesity, high blood pressure, high cholesterol, excessive alcohol consumption and physical inactivity, suggesting that the majority of cases are preventable [2].

In England, the National Health Service Health Check (NHSHC) provides a unique opportunity to target many CVD risk factors [2]. Introduced in 2009 by the Department of Health [3], the programme involves inviting everyone aged 40 to 74 years (who has not previously been diagnosed with CVD) to attend a NHSHC every 5 years, where their risk of heart disease, stroke, kidney disease and diabetes is assessed. The aims of this population prevention programme are (i) to reduce the risk of CVD in the population and subsequently the incidence of CVD events by providing individuals with support to make behavioural changes that will prevent the development of CVD, (ii) to facilitate early diagnosis of conditions such as high blood pressure, high cholesterol and type 2 diabetes and ensure that individuals get appropriate clinical management where needed, and (iii) to reduce inequalities in CVD health. In addition, people aged 65–74 are informed of the signs and symptoms of dementia, and sign-posted to memory clinics where appropriate [4]. A recent study highlighted the value of the NHSHC programme by demonstrating that patients who attend their NHSHC show demonstrable reductions in BMI, blood pressure and smoking incidence for 6 years afterwards [5], corroborating an earlier review which found that the programme can achieve small reductions in CVD risk [6].

Since April 2013, implementation of the programme has been the responsibility of local government (LG). Flexibility is permitted regarding how the programme is commissioned, although the tests, measurements and actions taken at certain risk factor thresholds are standardised to help ensure the safety, quality and effectiveness of the programme [4]. Substantial variation in programme delivery exists across LG, from the invitation process to the location of the checks (e.g., General Practitioner [GP] surgery, pharmacy etc.) to the healthcare professional carrying out the NHSHC.

Despite considerable variation in uptake rates across LG [7], there is little evidence for how these rates are influenced by differences in local programme delivery. This is an important area of focus given that ensuring a high percentage of those offered a NHSHC actually receive one is key to optimising the clinical and cost effectiveness of the programme [8]. Whilst there are no set targets for uptake, NHSHC funding allocations were originally modelled on an estimated uptake rate of 75% [8]. The national average uptake rate is currently 48.4% which means that just over half of invited patients do not attend their NHSHC [7]. Despite efforts, uptake of the NHSHC remains below optimum levels.

At present, the use of an invitation letter is the most common route for inviting eligible individuals for an NHSHC [9], and a standard template letter exists [7]. However, it is unclear how effective this method is compared to other invitation methods, and whether invitation mode varies in effectiveness for different groups of people. A review on general health checks [10] found that those least likely to attend were men on low incomes, those of low socio-economic status, the unemployed and the less well educated. Non-attenders also had a greater proportion of cardiovascular risk factors than attenders [10]. It is important to understand whether this pattern of attendance also holds for the NHSHC, and whether different groups of people are more likely to respond to certain interventions and invitation methods than others. The aim of this systematic review, therefore, is to highlight interventions and invitation methods that increase the uptake of NHSHCs, and to identify whether the effectiveness of these interact with broader patient and contextual factors. Literature that investigated the impact of patient demographic and contextual factors, but did not explore the impact of invitation methods or interventions, was also consulted in order to understand wider trends in uptake and help to interpret the findings of this review. To the authors’ knowledge there has been no systematic review published on this topic. Rapid reviews on similar topics have been completed by Cooper and Dugdill [11] and Usher-Smith and colleagues [6]. However the current paper is the first systematic literature review to report only high-quality evidence on interventions, invitation methods and patient and contextual characteristics that influence uptake of NHSHCs. In addition, no previous reviews have attempted to examine whether invitation methods and interventions vary in effectiveness by patient demographic characteristics. The overarching aim of conducting this systematic review is to contribute to evidence-based practice by translating evidence into current practice service delivery, and help steer the future direction of research.

## Method

The Preferred Reporting Items for Systematic Reviews and Meta Analyses (PRISMA) 27 item checklist [12] and the Critical Appraisal Skills Programme Systematic Review checklist [13], were used to structure and scrutinise the systematic review. This systematic review was registered with PROSPERO on 22/02/2016 (CRD42016035626). The original aim specified in the protocol was to identify interventions and invitation methods in hard-to-reach groups specifically. Due to the lack of studies focused on the uptake of NHS HCs in hard to reach groups, it was decided to expand the focus of this systematic review to include all those eligible for an NHS HC and explore the patient characteristics associated with uptake.

### Study eligibility

#### Inclusion criteria

The Patient-Intervention-Comparison-Outcome-Study (PICOS) framework [14] was used to develop eligibility criteria for the literature search strategy:

#### Patients

Eligible for a NHSHC (patients aged between 40 and 74 years with no existing diagnosis of heart disease, stroke, diabetes, kidney disease or high blood pressure).

#### Intervention

All studies that provided a clear description of the local implementation of the programme plus at least one of (i) the patient and or practice characteristics, (ii) invitation process, or (iii) an intervention implemented to encourage attendance at an NHSHC were included.

#### Comparison

Standard invitation method (for intervention studies), other types of invitation method (for studies comparing different existing methods if invitation), patients who do not attend NHSHCs (for studies investigating patient demographic characteristics).

#### Outcome

Uptake of or attendance at the NHSHC.

### Study type and design

Only studies focused on NHSHCs were included in this review. Studies were required to have been published in 2009 onwards (as this is when the programme was implemented) and in English (as this is the only language spoken by the research team). This review intended to exclusively include randomised controlled trials since they are most able to support inferences of causality about interventions, but due to the limited number of studies, quasi-experimental research design trials were also included. Therefore, the following study designs were included: randomised controlled trials, observational cohort studies or cross-sectional studies, which may also be used to support inferences about causality.

#### Exclusion criteria

Any studies that were qualitative in design, a service evaluation or reported only subjective or self-reported outcomes were excluded. Any studies focused on children or individuals previously diagnosed with CVD or any interventions that focused on screening or disease-specific health checks other than NHSHC were excluded.

### Search strategy

Between January 2015 and May 2015, a systematic review was conducted. This was repeated in August 2016 and then again in March 2018. A different list of databases was searched in March 2018 as a result of the lead author’s completion of a university course and thus the termination of access to university library services (Table 1).

**Table 1:** Databases searched in 2016 and 2018

Searches up to August 2016	March 2018
• Cochrane Library (Including Cochrane Database of Systematic Reviews and Cochrane Central Register of Controlled Trials) • EBSCOHOST (Including CINAHL Plus with full text, Psych Info, Psych Articles and MEDLINE) • Ovid (including Embase) • SCOPUS • Web of Science • Google Scholar	• Ovid Medline • Ovid Embase • Ovid Psycinfo • EBSCO CINAHL • CDSR and CTR • SCOPUS • Google Scholar

The Cochrane Library, Database of Abstracts of Reviews of Effects (DARE), Trip Database, NICE Evidence Services and PubMed Health were also searched at each time point to identify relevant systematic reviews. The reference list of review articles and all studies included within the review were also searched in order to find other potentially eligible studies. A hand search was carried out in recent journal editions.

The searches included terms selected to identify literature that (i) was relevant to the NHSHC specifically (e.g., “NHS Health Check” OR health check* OR (nhs and health check*)), (ii) investigated the impact of invitation methods and interventions (e.g., intervention* OR invit* OR offer* OR encourage*) and (iii) included uptake as an outcome measure (e.g., uptake OR attend* OR appointment*). See Additional file 1 for the full list of search terms used in this review and Additional file 2 for the hits by database.

### Study selection

**Fig. 1:**
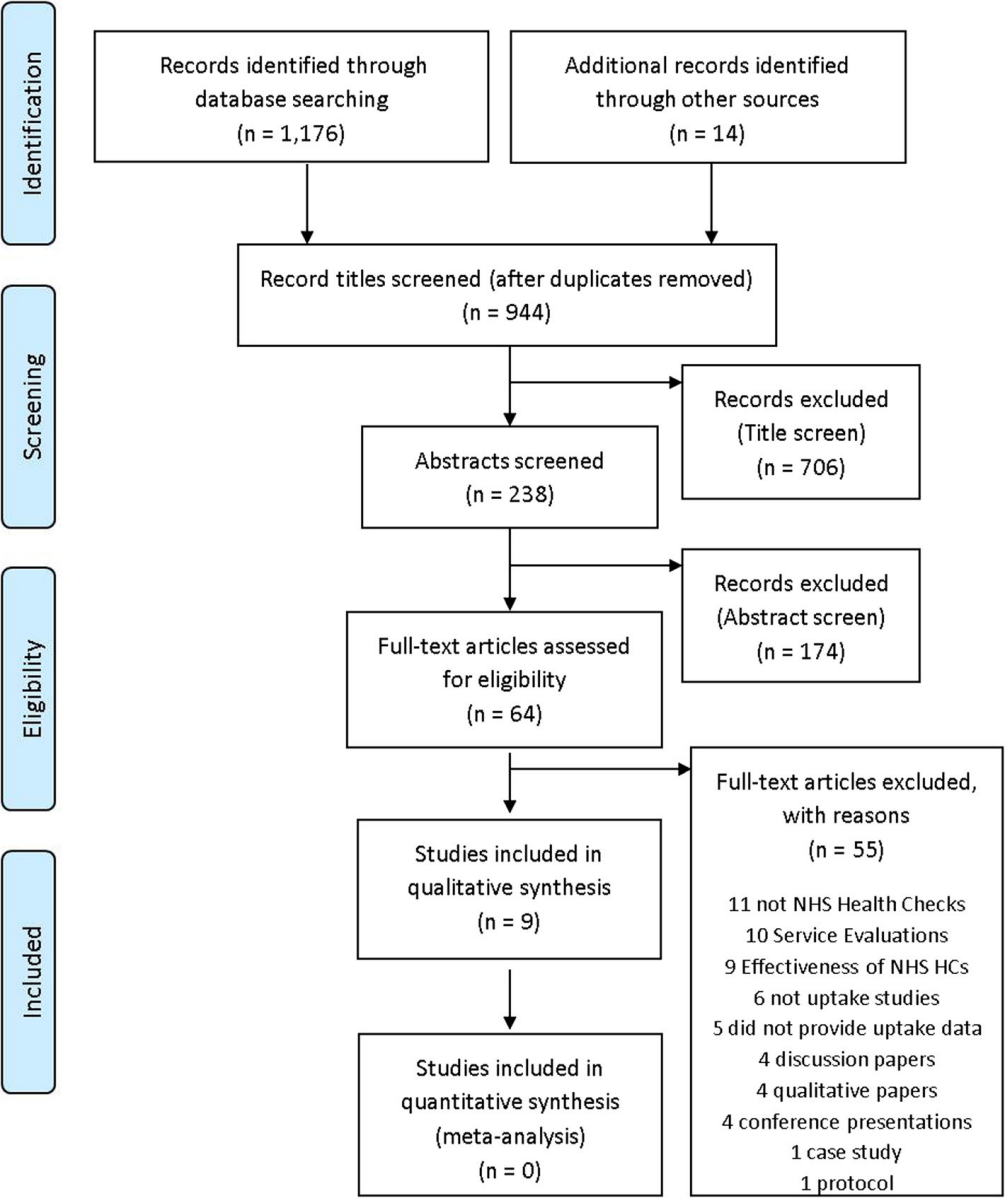
Flow diagram of review process using the PRISMA (2009) template

Study records (titles) were screened by one researcher (AKB 2015 & 2016; LP 2018) in EndNote to identify articles for detailed abstract screening. One researcher (AKB 2015 & 2016; LP 2018) selected suitable abstracts (or those which did not provide sufficient information for eligibility assessment) for full review. Full review involved two researchers (AKB & BH 2015 & 2016; AKB & LP 2018) screening the full study text and populating a data extraction form to ensure the study met the inclusion criteria (Additional file 3). Figure 1 details the number of articles assessed at each stage. A list of studies excluded at the full text review stage and justifications is included in Additional file 4. The nine studies that met the inclusion criteria also underwent a quality assessment using an adapted tool (Additional file 5) specially developed to accommodate both randomised and non-randomised studies. Two researchers reviewed the quality of selected studies independently (AKB & BH 2015 & 2016; AKB & LP 2018) The checklist scores were reviewed, and then any discrepancies were identified discussed and resolved by referring to the research paper.

For the initial 2015 and 2016 reviews (in which seven papers were identified as eligible for inclusion) the interrater reliability was calculated using Cohen’s kappa [15], which showed substantial agreement between the raters (k = 0.772, *p* < 0.0005) [16]. The 2018 reviewers were in 100% agreement over the additional two papers identified.

### Analysis

A narrative synthesis is used to present the findings of this systematic review using the guidance from the Economic and Social Research Council [17]. A meta-analysis was not planned and has not been conducted as pooling results obtained from diverse non-randomised study-designs is not recommended [18].

## Results

### Search results

Nine-hundred-and-forty-four studies were identified after performing electronic de-duplication within and between each database (Additional file 2). After initial title screening, 238 titles and abstracts were identified as potentially relevant, of which 64 full papers were retrieved and assessed for eligibility (Fig. 1). A total of nine papers were included in the final review. No further studies were identified through the reference lists of the papers included, or subsequent hand searching.

### Study Characteristics & Methodological Quality

The nine included studies were conducted between 2011 and 2016, using data from 2008 to 2014. The sample sizes varied from 1380 patients [19] to 40,112 patients [20], and included between four [19, 21] and 38 GP Practices [22]. The length of studies (including the follow up period) varied from 6 months [23] to 4 years [22]. Six studies achieved a strong quality assessment rating [9, 20, 21, 23–25] and three achieved a moderate rating [19, 22, 26]. There were only two randomised controlled trials identified [21, 25], with the remaining studies consisting of one observational cohort [9] and six cross sectional studies [19, 20, 22–24, 26]. Table 2 summarises the study characteristics.

**Table 2:** Study characteristics

Author	Design	Time Period	Setting	Participants	Factors Investigated	Funding Sources Acknowledged	Quality Score
Artac et al., (2013) [20]	Cross-sectional	2008–2011	GP Practices within Hammersmith and Fulham primary care trust, London. Twenty-seven practices in Year 1, 29 practices in Year 2	Year 1 4748 high risk patients (mean age 60.9 years, 78.4% male) Year 2 35,364 patients eligible for the NHSHC (mean age 50.0 years, 45.2% male)	Demographic factors – age, gender, deprivation, ethnicity, risk factors, practice	NIHR North West London Collaboration for Leadership in Applied Health Research; NHS Hammersmith and Fulham; Department of Health Policy Research Programme	19 (Strong)
Attwood et al., (2016) [19]	Cross-sectional	Not given	Four GP Practices in the East of England	1380 patients invited to the NHSHC (mean age 52.4 years, 49.7% male)	Demographic factors – age, gender, deprivation, ethnicity, practice	British Heart Foundation, Cancer Research UK, ESRC, MRC, NIHR, Wellcome Trust	20 (Moderate)
Cochrane et al., (2013) [23]	Cross-sectional	August 2009 – January 2010	Thirty-seven GP Practices in Stoke-on-Trent	10,483 patients invited to the NHSHC (aged 32–74 years, no mean age given; 81.3% male. Patients aged from 32 years were included due to above-average burden of CVD in the area and below-average life expectancy).	Demographic factors – age, gender, deprivation, risk level, practice	Stoke-on-Trent Primary Care Trust	19 (Strong)
Coghill et al., (2018) [22]	Cross-sectional	June 2010 – October 2014	Thirty-eight GP Practices in Bristol	31,881 patients invited to the NHSHC (mean age 52.4 years, 52% male)	Demographic factors – age, gender, deprivation	NIHR School for Primary Care Research; Public Health Bristol; NIHR Collaboration for Leadership in Applied Health Research West	20 (Moderate)
Cook et al., (2016) [26]	Cross-sectional	April 2013 March 2014	Thirty GP Practices in Luton	13,063 patients invited to the NHSHC (no mean age given; 53.3% male)	Invitation method; invitation method by ethnicity and gender Demographic factors – age, gender, deprivation, ethnicity	No funding stated	23 (Moderate)
Dalton et al., (2011) [24]	Cross-sectional	September 2008 – January 2010	Twenty-nine GP Practices in Ealing, London	5294 patients invited to the NHSHC (aged 35–74 years, no mean age given. Patients from the age of 35 were invited in this study due to earlier onset of CVD and diabetes in the local area. 80.9% male)	Demographic factors – age, gender, deprivation, ethnicity, risk factor, practice	NHS Ealing; Higher Education Funding Council for England; NIHR Collaboration for Leadership in Applied Health Research & Care	19 (Strong)
Gidlow et al., (2014) [9]	Observational cohort	September 2010 – February 2014	Five GP Practices in Stoke-on-Trent	4855 patients invited to the NHSHC (mean age 53.4 years, 46.9% male)	Invitation method Demographic factors – age, gender, deprivation, ethnicity, practice	Stoke-on-Trent Public Health Directorate	19 (Strong)
McDermott et al., (2018) [25]	Randomised controlled trial (three arms)	July 2013 – December 2014	Eighteen GP Practices in Lambeth and Lewisham, London	12,459 patients invited to the NHS HC (median age 45; 52% male)	Intervention (sending a Question Behaviour Effect questionnaire ahead of invitation vs. QBE questionnaire plus incentive to return questionnaire vs. control) Demographic factors – age, gender, deprivation, ethnicity	NIHR Health Technology Assessment Programme; Guy’s and St Thomas’ Charity; NIHR Biomedical Research Centre; Cancer Research UK	16 (strong)
Sallis et al., (2016) [21]	Randomised controlled trial (two arms)	2013–2014	Four GP Practices in Medway	3511 patients invited to the NHSHC (control arm mean age 53.1 years, 46.5% male; intervention arm mean age 52.8 years, 49.1% male)	Intervention (enhanced letter vs. standard letter) Demographic factors – age, gender, deprivation, practice	Department of Health	16 (strong)

### NHSHC uptake

#### Invitation methods

Two studies found that oral invitations (e.g., telephone invitations from the practice or opportunistic face-to-face invitations at the practice) were more effective than written letter invitations, despite letters being the most commonly employed invitation type. Gidlow and colleagues [9] found that the majority of the five GP practices that they studied usually invited eligible patients to attend via an invitation letter (72% of patients were invited in this way) but that the odds of individuals attending were three times greater when they were invited using a telephone/face-to-face approach compared to by letter alone (OR 2.87, 95% CI =2.26–3.64). Similarly, Cook and colleagues [26] found that letters were the most common form of invitation but that face-to-face invitations had the highest overall uptake rate of 71.9% with uptake rates for both telephone (43%) and letter (29.5%) invitations markedly lower.

Only one study investigated whether invitation method effectiveness differed according to patient characteristics (although it is important to note that this was a cross-sectional study and did not randomise patients to different invitation methods) [26]. Given that letters were the most common method of invitation across the practices included in the study, it is unsurprising that those groups who had the lowest uptake rates overall also showed some of the lowest uptake rates for letter invitations specifically (e.g., ‘Any Other White Background’ males uptake = 19%, *p* < .001, ‘Any Other White Background’ females uptake = 22%, *p* < .001, and ‘African’ females *p* = 23%, *p* < .050). Face-to-face invitations were found to be most effective for ‘White Irish’ females (uptake = 93%, p < .050) and ‘White British’ male (uptake = 72%, *p* < .001) and female patients (uptake = 70%, *p* < .001), but the least effective method for inviting ‘Bangladeshi’ (uptake = 43%, *p* < .001) and ‘Pakistani’ males (uptake = 47%, *p* < .050). Invitation by telephone was the least common method; however, where this method was used, it was most effective for ‘Pakistani’ patients of both genders (uptake = 100%, *p* < .010 for both genders), ‘White/Black Caribbean’ females (uptake = 100%, *p* < .001), ‘White Irish’ females (uptake = 96%, *p* < .001) and ‘Asian (Other)’ females (uptake = 76%, *p* < .001), but least effective for ‘White British’ females (uptake = 0%, *p* < .001) and ‘Any Other White Background’ patients (uptake of 10 and 8% for males and females respectively, both *p* < .001).

#### Interventions on invitation methods

Two studies were identified that tested interventions to enhance invitation methods with varying success. Sallis and colleagues [21] enhanced invitation letters using techniques from behavioural science including simplifying the text, personalisation, emphasising timelines (i.e., that the NHSHC was “due”) and providing patients with a space to write down the time and date of their appointment. The odds of attending a NHSHC appointment were 26% higher for patients receiving an enhanced invitation letter compared to patients receiving the control letter (AOR = 1.26, 95% CI = 1.09–1.47, *p* < .001). Uptake in the intervention arm was 33.5% compared to 29.3% in the control arm, an absolute difference of 4.2% and a relative difference of 14.3% in NHSHC attendance. However, McDermott and colleagues [25] found that mailing patients a Question-Behaviour Effect questionnaire (with or without an incentive for returning the questionnaire) 1 week before invitation letters did not significantly improve uptake in the intervention groups compared to the control group. These researchers found that uptake was higher for intervention group individuals who returned the questionnaire, but when examining the intervention group as a whole (i.e., in an intention to treat analysis), there was no significant effect of including a questionnaire (*p* = .070) or a questionnaire plus incentive (*p* = .054) compared to controls. Less than a quarter of participants returned the questionnaire and those who returned the questionnaires were more likely to be female, in older age groups, and have lower levels of deprivation. Interestingly, all of these demographic factors are associated with higher uptake of health checks which is discussed in the following section. Neither of the trials investigating interventions to improve uptake explored whether the effectiveness of the interventions interacted with patient demographic characteristics.

### Socio-demographic factors on uptake of NHSHCs

#### Age

All studies found that older patients were more likely to attend than younger patients (Table 3). This finding was consistent, regardless of whether studies tested the effects of age in increments of years [9, 19] or decades [21], or whether broader age groups were tested [20]. In addition, Dalton and colleagues found a significant interaction between age and gender, with women in the youngest age-group (35–54 years) more likely to attend than men of the same age-group [24].

**Table 3:** Study findings on impact of age on NHSHC uptake. AOR = adjusted odds ratio. OR = odds ratio. CI = confidence intervals. UR = uptake rate

Study	Findings
Artac et al., 2013 [20]	55–64 age-group vs. 40–54 age-group (baseline): AOR = 1.34, 95% CI = 1.11–1.61, *p <* .050 65–74 age-group vs. 40–54 age-group (baseline): AOR = 2.05, 95% CI = 1.67–2.52, *p <* .010
Attwood et al., 2016 [19]	5% increase in likelihood of uptake with each additional year in age: OR = 1.05, 95% CI = 1.04–1.07, *p <* .010
Cochrane et al., 2013 [23]	Overall effect of age-group: OR = 1.64, 95% CI = 1.51–1.77, *p <* .001
Coghill et al., 2018 [22]	Overall effect of age *p <* .001 50–59 years vs. ≤ 49 years (baseline): AOR = 1.36, 95% CI = 1.21–1.53 60–69 years vs. ≤ 49 years (baseline): AOR = 2.19, 95% CI = 1.80–2.68 70+ years vs. ≤ 49 years (baseline): AOR = 2.53, 95% CI = 1.89–3.39
Cook et al., 2016 [26]	Highest uptake in 65–69 (male uptake = 71%, *p* < .001, female uptake = 62%, *p <* .001) and 70–74 age-groups (male uptake = 68%, *p* < .001, female uptake = 80%, *p* < .001)
Dalton et al., 2011 [24]	55–64 age-group vs. 35–54 age-group (baseline): AOR = 1.74, 95% CI = 1.34–2.25, *p* < .001 65–74 age-group vs. 35–54 age-group (baseline): AOR = 2.27, 95% CI = 1.47–3.50, *p* < .001 Significant age x gender interaction; women in the youngest age-group (35–54 years) more likely to attend than men in the same age category: AOR = 1.71, 95% CI = 1.03–2.85, *p* = .037
Gidlow et al., 2014 [9]	4% increase in likelihood of uptake with each additional year of age: OR = 1.04, 95% CI = 1.03–1.04, *p* < .001
McDermott et al., 2018 [25]	60–74 age group vs. 40–59 age-group (baseline): OR = 1.43, 95% CI = 1.20–1.71, *p* < .001
Sallis et al., 2016 [21]	62% increase in likelihood of uptake with every additional 10 years in age: AOR = 1.62, 95% CI = 1.50–1.75, *p* < .010

#### Gender

The majority of studies found that uptake was highest for female patients (Table 4). Two studies both found that female patients were 50% more likely to attend than male patients [19, 21] (although one of these found that this association was no longer significant once practice had been added to the adjusted model [19]) while another found a similar increase of 47% likelihood for female patients [9]. Artac and colleagues found higher uptake among females when examining data from Year 2 when all eligible patients were invited for a NHSHC, but no gender difference was found in Year 1, when only high-risk patients were invited, 78.4% of whom were male [20]. In contrast to the majority of studies, Cochrane and colleagues [23] found lower uptake amongst female patients. Finally, as noted earlier, another study found that there was a significant interaction effect between age and sex, with women in the youngest age group being more likely to attend the NHSHC than men [24].

**Table 4:** Study findings on impact of gender on NHSHC uptake. AOR = adjusted odds ratio. OR = odds ratio. CI = confidence intervals.

Study	Findings
Artac et al., 2013 [20]	Year 1 (high-risk only) Female vs. male (baseline): AOR = 0.80, 95% CI = 0.67–0.94 *p* < .010 Year 2 (all eligible patients) Female vs. male (baseline): AOR = 1.27, 95% CI = 1.20–1.35, *p* < .010
Attwood et al., 2016 [19]	Unadjusted model Female vs. male (baseline): OR = 1.50, 95% CI = 1.16–1.95, *p* < .050 Model adjusted for GP surgery effects Female vs. male (baseline): AOR = 1.29, 95% CI = 0.95–1.76, *p* > .050
Cochrane et al., 2013 [23]	Female vs. male (baseline): OR = 0.70, 95% CI = 0.58–0.84, *p* < .001
Coghill et al., 2018 [22]	Male vs. female (baseline): AOR = 0.73, 95% CI = 0.67–0.80, *p* < .001
Cook et al., 2016 [26]	Female uptake rate = 50%; male uptake rate = 38%, *p* < .001
Dalton et al., 2011 [24]	Significant age x gender interaction; women in the youngest age-group (35–54 years) more likely to attend than men in the same age category: AOR = 1.71, 95% CI = 1.03–2.85, *p* = .037
Gidlow et al., 2014 [9]	Female vs. male (baseline): OR = 1.47, 95% CI = 1.30–1.68, *p* < .001
McDermott et al., 2018 [25]	Male vs. female (baseline): AOR = 0.74, 95% CI = 0.69–0.80, *p* < .001
Sallis et al., 2016 [21]	Female vs. male (baseline): AOR = 1.50, 95% CI = 1.29–1.74, *p* < .010)

#### Deprivation

Where a significant effect of deprivation on uptake was found, the majority of studies reported that this was due to lower uptake in more deprived groups ([9, 21, 25, 26]; see Table 5). However, in other studies, this relationship was dependent on whether analyses were adjusted for other factors or not. For example, in unadjusted analyses, Cochrane and colleagues [23] found a significant pattern of decreasing uptake as practice-level deprivation increased. However, deprivation was no longer significant when analyses were adjusted for gender, age, risk category and practice size.

Meanwhile, Attwood and colleagues [19] found that the direction of the relationship between deprivation and uptake depended on whether or not analyses were adjusted for other predictors (e.g., age, gender, ethnicity, GP practice), with adjusted analyses revealing the same pattern of lower uptake in the most deprived groups as seen in many other studies, but unadjusted analyses revealing *higher* uptake in more deprived groups. Similarly, in adjusted analyses, Artac and colleagues [20] found higher uptake amongst patients living in deprived areas in Year 2 of the programme only (when all eligible patients were invited; no effect of deprivation was found when only high-risk patients were invited). Two studies [22, 24] found no significant effect of deprivation on uptake.

**Table 5:** Study findings on impact of deprivation on uptake. Quintile/tertile 1 refers to the most deprived group. Note that some studies [19, 22, 25, 26] coded deprivation so that quintile/tertile 1 referred to the least deprived group, but that this has been reversed for the current narrative synthesis in order to match other studies’ reporting standards and enhance comparability across studies. IMD = Index of Multiple Deprivation, AOR = adjusted odds ratio. OR = odds ratio. CI = confidence intervals

Study	Findings
Artac et al., 2013 [20]	Year 1 analyses (high-risk patients only) IMD Tertile 3 vs. 1: AOR = 0.84, 95% CI = 0.69–1.01, *p* > .050 IMD Tertile 2 vs. 1: AOR = 0.94, 95% CI = 0.79–1.13, *p* > .050 Year 2 analyses (all eligible patients) IMD Tertile 3 vs. 1: AOR = 0.80, 95% CI = 0.73–0.87, *p* < .010 IMD Tertile 2 vs. 1: AOR = 0.84, 95% CI = 0.78–0.90, *p* < .010
Attwood et al., 2016 [19]	Unadjusted analyses IMD Quintile 2 vs. 5: OR = 2.17, 95% CI = 1.39–3.38, *p* < .010 IMD Quintile 1 vs. 5: OR = 2.90, 95% CI = 1.84–4.58, *p* < .010 Adjusted analyses IMD Quintile 2 vs. 5: AOR = 0.37, 95% CI = 0.18–0.67, *p* < .050 IMD Quintile 1 vs. 5: AOR = 0.42, 95% CI = 0.20–0.88, *p* < .050
Cochrane et al., 2013 [23]	Lowest attendance in tertile 3 (attendance rate = 42.6%, *p* < .050) Highest attendance in tertile 1 (attendance rate = 48.4%, *p* < .050 Deprivation was no longer significant when analyses were adjusted for gender, age, risk category and practice size AOR = 1.12, 95% CI = 0.96–1.30
Coghill et al., 2018 [22]	Non-significant effect of deprivation on uptake (*p* = .053)
Cook et al., 2016 [26]	Lowest uptake in Quintile 1 with uptake rates of 0.31 and 0.38 for males and females respectively, *p* < .001 Highest uptake in the Quintile 5, with uptake rates of 0.53 and 0.60 respectively, *p* < .001
Dalton et al., 2011 [24]	No significant effect of deprivation (*p* > .050)
Gidlow et al., 2014 [9]	IMD Quintile 5 vs. 1: OR = 1.59, 95% CI = 1.23–2.05, *p* < .001 IMD Quintile 4 vs. 1: OR = 1.30, 95% CI = 1.06–1.61, *p* = .014 IMD Quintile 3 vs. 1: OR = 1.24, 95% CI = 1.03–1.49, *p* = .022 IMD Quintile 2 vs. 1: OR = 1.11, 95% CI = 0.87–1.43, *p* = .395 Overall effect of deprivation *p* = .008
McDermott et al., 2018 [25]	IMD Quintile 4 vs. 1: AOR = 2.78, 95% CI = 1.87–4.12, *p* < .001 IMD Quintile 3 vs. 1: AOR = 1.15, 95% CI = 0.95–1.39, *p* = .156 IMD Quintile 2 vs. 1: AOR = 1.09, 95% CI = 0.95–1.24, *p* = .214 (Note, no data was collected from Quintile 5 in this study)
Sallis et al., 2016 [21]	IMD Quintile 5 vs. 1: AOR = 1.61, 95% CI = 1.14–2.26, *p* < .010 All other comparisons against Quintile 1 (baseline) *p* > .010

#### Ethnicity

Findings on ethnicity presented a mixed picture across the studies: some found that attendance was significantly higher in certain ethnic groups and others found that uptake did not differ by patient ethnicity. Uptake was found to be higher in patients from Asian groups (including South Asian and Asian-Indian groups) [20, 24–26]; Black groups (including Black African and Black Caribbean groups) [20, 25]; and mixed ethnicity groups [24, 25]. Contrary to other findings, Cook and colleagues [26] found that amongst females, uptake rates were lowest for Black African patients, however they observed higher uptake rates amongst Black Caribbean patients of both genders in line with other studies reporting high uptake amongst Black patients. In contrast, two studies found that there was no significant difference by ethnic group [9, 19]. Detailed findings on ethnicity are presented in Table 6 below.

**Table 6:** Summary of findings on ethnicity across studies. AOR = adjusted odds ratio. OR = odds ratio. CI = confidence intervals. UR = uptake rate

Study	Findings
Artac et al., 2013 [20]	In Year 2, higher in patients of South Asian (AOR = 1.50, 95% CI = 1.25–1.78, *p* < .010) and Black ethnicity (AOR = 1.58, 95% CI = 1.43–1.75, *p* < .010) and Others (AOR = 1.16, 95% CI = 1.07–1.25, *p* < .010), where White is baseline.
Attwood et al., 2016 [19]	No difference between White ethnicity vs. Other ethnicity (OR = 0.59, 95% CI = 0.21–1.57)
Cook et al., 2016 [26]	Higher uptake amongst Mixed White and Asian males (UR = 0.91, 95% CI = 0.66–0.99, *p* < .001), Caribbean males (UR = 0.69, 95% CI = 0.62–0.76, *p* < .001),Chinese males (UR = 0.67, 95% CI = 0.45–0.84, *p* < .010), Chinese females (UR = 0.93, 95% CI = 0.72–0.99, *p* < .001), White/Black Caribbean females (UR = 0.77, 95% CI = 0.63–0.87, *p* < .001), White Irish females (UR = 0.72, 95% CI = 0.64–0.80, *p* < .001), and Black Caribbean females (UR = 0.71, 95% CI = 0.64–0.77, *p* < .001). Lower uptake amongst any other white patients (male UR = 0.27, 95% CI = 0.24–0.30, *p* < .001; female UR = 0.35, 95% CI = 0.31–0.38, *p* < .001) and Black African females (UR = 0.42, 95% CI = 0.37–0.47, *p* < .010).
Dalton et al., 2011 [24]	When compared to White British patients, uptake higher in patients of South Asian (AOR = 1.71, 95% CI = 1.29–2.27, *p* < .001) and mixed ethnic backgrounds (AOR = 2.42, 95% CI = 1.50–3.89, *p* = .015).
Gidlow et al., 2014 [9]	No difference between White, Mixed, Asian, Black or Other ethnicity groups (X^2^ = 0.769, *p* = .380)
McDermott et al., 2018 [25]	Higher uptake amongst Asian (OR = 2.03, 95% CI = 1.63–2.67, *p* < .001), African/Caribbean (OR = 2.15, 95% CI = 1.86–2.49, *p* < .001) and mixed (OR = 3.09, 95% CI = 2.07–4.62, *p* < .001) ethnicity groups compared to White patients.

#### Medical and lifestyle risk

Studies’ conceptualisation of risk varied, with some classifying it as a medical risk (e.g., family history of CVD) and others including lifestyle factors (e.g., smoking status; see Table 7). Cochrane and colleagues [23] found a non-significant trend towards decreased likelihood of attendance for patients deemed at higher risk of CVD. Conversely, Artac and colleagues found that risk factors such as presence of non-CVD comorbidities and family history of coronary heart disease were both significant predictors for increased uptake across both years of the programme, whereas smoking status was a significant predictor for decreased uptake across both years [20]. A non-significant trend for lower uptake amongst smokers was found by Dalton and colleagues [24]. This suggests that the association between risk level and uptake may vary depending on the definition of risk and whether risk is assessed based on medical history or lifestyle factors (Table 7).

**Table 7:** Study findings on impact of risk factors on NHSHC uptake. AOR = adjusted odds ratio. OR = odds ratio. CI = confidence intervals. UR = uptake rate

Study	Findings
Artac et al., 2013 [20]	Impact of comorbidities (medical risk) Year 1 AOR = 1.53, 95% CI = 1.31–1.80, *p* < .010 Year 2 AOR = 1.75, 95% CI = 1.64–1.87, *p* < .010 Impact of family history (medical risk) Year 1 AOR = 2.49, 95% CI = 2.15–2.90, *p* < .010 Year 2 AOR = 2.01, 95% CI = 1.87–2.16, *p* < .010 Impact of smoking status (lifestyle risk) Year 1 AOR = 0.71, 95% CI = 0.61–0.83, *p* < .010 Year 2 AOR = 0.83, 95% CI = 0.77–0.90, *p* < .010)
Cochrane et al., 2013 [23]	Risk category (combination of medical and lifestyle risk) OR = 0.90, 95% CI = 0.80–1.02, *p* > .050 and < .100
Dalton et al., 2011 [24]	Smoking status (lifestyle risk) Yes vs. no (baseline): AOR = 0.88, 95% CI = 0.75–1.92, *p* = .097

#### Practice differences

Interestingly, all studies reporting on GP practice found significant variance in uptake between practices (Table 8) [9, 19–21, 23, 24], however, it was not always possible to discern the reasons for this. Two studies found some evidence that practice list size impacted NHSHC attendance [20, 24], however the direction of these effects was different, and Cochrane and colleagues [23] found that practice size was not significantly related to uptake. Gidlow and colleagues [9] found that NHSHC attendance did not vary by distance to practice from the patient’s home. No other specific practice-level factors were reported.

**Table 8:** Study findings on the impact of practice and specific practice-level factors on NHSHC uptake. AOR = adjusted odds ratio. OR = odds ratio. CI = confidence intervals. UR = uptake rate

Study	Findings
Artac et al., 2013 [20]	Practice List Size Year 2 (all eligible patients) > 10,000 vs. < 6000 (baseline): AOR = 6.05, 95% CI = 0.84–43.3, *p* < .010 Unexplained variance in models was interpreted as attributable to unmeasured practice factors Year 1 unexplained variance = 19.4, 95% CI = 15.2–24.4% Year 2 unexplained variance = 37.3, 95% CI = 30.6–44.6%
Attwood et al., 2016 [19]	Significant variance in uptake by practice *X*^*2*^_*2*_ = 74.61, *p* < .005 Adjusting analyses for GP Practice had a substantial effect on the strength and direction of associations between socio-demographic variables (specifically gender and IMD quintile; see relevant sections above) and uptake.
Cochrane et al., 2013 [23]	Practice Size AOR = 1.03, 95% CI = 0.88–1.20, *p* > .100 Variance accounted for by individual practices = 12.7%, *p* < .001
Dalton et al., 2011 [24]	Practice List Size < 3000 vs. 3000–5999 (baseline): AOR = 2.53, 95% CI = 1.09–5.84, *p* = .030 ≥ 6000 vs. 3000–5999 (baseline): AOR = 0.79, 95% CI = 0.33–1.88, *p* = .599 Variance in models accounted for by practice = 28% (VPC = 0.28)
Gidlow et al., 2014 [9]	Variation in uptake by practice *X*^*2*^ = 336.9, *p* < .001 Variation in uptake by distance to practice *X*^*2*^ = 0.478, *p* = .924
Sallis et al., 2016 [21]	Of the five practices studied, one (used as baseline in analyses) had significantly higher uptake rates than all others (all *p* < .010).

## Discussion

This review aimed to identify invitation methods and interventions that increased uptake of NHSHCs, and to explore whether the effectiveness of these varied by patient demographic characteristics and contextual factors. The studies included all achieved a high or moderate quality rating, suggesting that risk of bias is low. Overall, it was possible to identify which invitation methods and patient demographic characteristics were associated with increased uptake, but very little evidence was available with regards to how patient demographic characteristics interact with invitation methods and interventions to increase uptake, representing a significant limitation of the existing literature. Letters are the most widely used invitation method within the NHSHC programme [9, 26] and our findings revealed that compared to telephone and opportunistic face-to-face invitations, they were the least effective at encouraging uptake. Given this, it is perhaps unsurprising that the only two interventions identified in our search both focused on enhancing letter invitation techniques. Sallis and colleagues [21] made changes to the existing national template letter using a behavioural insights approach, and found an increase in uptake for those who received the intervention letter. Two recent studies (one under review, one published after our searches were conducted) also found significant, positive impacts of enhancing invitation letters using insights from behavioural science; one study used techniques such as message simplification and encouraging behavioural planning in the letter to increase uptake [27] whilst another enhanced uptake by either discussing sunk costs in the letter (telling patients that funding had already been set aside for their appointment) or providing counterarguments against common reasons for not attending (e.g., by telling patients that lifestyle factors can have an impact on CVD risk even in the presence of a family history of disease [28].

Meanwhile, McDermott and colleagues [25] found that posting a Question-Behaviour Effect questionnaire (with or without a financial incentive to encourage the questionnaire’s return) ahead of invitation letters did not have a significant effect on uptake, possibly due to low rates of questionnaire return. This technique has been successful in other areas (e.g., general health checks, influenza vaccinations [29], so its failure to increase NHS Health Check uptake highlights the importance of trialling interventions applied to new areas, even when they have been successful in similar fields. Recently, Gold and colleagues found that providing an enhanced leaflet alongside invitation letters also failed to significantly impact uptake rates [30]. Together, these findings suggest that making enhancements to the invitations directly (instead of to any accompanying materials) may be the most effective strategy to improve uptake.

The results of this review with regards to effectiveness of invitation method are reflected elsewhere in the literature. For example, a recent study (published outside of our search dates) also found that uptake was significantly higher after telephone invitations (estimated effect was an additional 180 NHSHCs attended for every 1000 patients) compared to letters personalised to patients’ CVD risk (estimated additional 40 NHSHCs per 1000 patients) and the standard invitation letter in use at the time [31]. Letters are a low-cost invitation method and have previously been recommended due to a lack of evidence for the cost-effectiveness of other methods [32]; however there is evidence that the high uptake rates associated with telephone invitations may make this method more cost-effective [31].

There are a couple of possible explanations for this relative effectiveness at encouraging uptake. A recent review and qualitative synthesis identified a number of reasons for not taking up the offer of a NHSHC, including misunderstanding the purpose of the appointment and the prioritisation of other daily demands [33]. It is possible that speaking directly to patients through telephone and face-to-face invitations allows these barriers to be discussed and broken-down, while also removing some of the steps in the appointment booking process. Recent work examining telephone outreach approaches revealed that patients appreciated being able to ask questions and receive immediate answers, and that patients also appreciated the immediacy of being able to book an appointment during the outreach call, stating that they might not have gotten around to booking an appointment or even reading the letter in the first place [34]. .However, it is also worth noting that since the studies reviewed here were conducted, the national template letter has been updated based on a series of studies that successfully increased NHSHC uptake by enhancing this letter [21, 27] (one of which was reviewed here [21];), meaning that comparisons of oral methods against written methods may yield different outcomes in future research.

The study by Cook and colleagues (which was the only one identified exploring invitation method effectiveness by demographic characteristics) found that the effectiveness of invitation methods varied by patient ethnicity (although it should be noted that this was not a randomised study and while it achieved a moderate rating in the quality assessment, it was only one point away from being scored as low quality) [26]. For example, whilst face-to-face invitations were most effective for White British patients, they were least effective for Bangladeshi and Pakistani males; conversely, telephone invitations were most effective for Pakistani patients and least effective for White British females and those identifying as any other white ethnicity. While the reasons for this differential success were not explored in the study by Cook and colleagues [26], a recent qualitative investigation of a telephone outreach intervention for deprived communities and ethnic minority groups found that patients appreciated receiving a proactive invitation by telephone, and particularly valued calls when the caller was someone with whom they could culturally identify and, in cases of language barriers, communicate in their first language [34]. This corroborates qualitative evidence from other health programmes which found that difficulties with reading written materials in English posed a barrier for South Asian patients [35]. It is not clear whether the GP practices included in the study by Cook and colleagues contacted patients through a similar outreach programme targeted towards ethnic minority groups, however these findings provide a starting point both for future research and current practice. In particular, where telephone/face-to-face invitations may not be feasible or affordable for practices to deliver to all eligible patients, identifying specific patient groups (i.e., those who are high risk or who are likely to be particularly responsive to this type of invitation) for telephone invitations could be an appropriate strategy for allocating resources effectively.

An alternative use of resources could be to use telephone invitations as a follow-up reminder for patients who do not respond to the initial letter; evidence from increasing attendance at hospital appointments has shown the effectiveness of reminder messages [36], suggesting that reminder phone calls could be a successful strategy. Research in this area is also testing the effectiveness of text messages, which are cheaper per patient to send than phone calls; one study found that sending a reminder text as a follow-up to a letter increased NHSHC uptake [27]. These results show how different forms of telephone contact can improve NHSHC uptake. Future research could therefore investigate the cost-effectiveness of text message reminders as a method to engage those groups with lowest attendance rates. Research could also investigate if these methods could minimise the difference in attendance by demographic factors, or whether logistical issues (e.g., related to keeping updated records of patient phone numbers) would limit the effectiveness of this method.

In terms of the impact of demographic factors on uptake, the review revealed that all studies found higher NHSHC attendance in older patients and the majority of studies found that females were more likely to attend than males. This latter finding is concerning as men may be at greater risk of CVD than women (for example, over 70% of high-risk patients were male in one study [20]). However, there was some variation, with two studies finding that male patients were more likely to attend than females, and another finding that the beneficial effect of being female was no longer significant once GP surgery had been accounted for in the model, suggesting that the effect of gender on uptake is not as reliable as the effect of age. As suggested by Usher-Smith and colleagues [6], the finding of an interaction between age and gender in one study (where females in the youngest age category were more likely to attend than males, but no difference was detected for older patients) [24] may provide an explanation for this variability across studies, in that younger female patients are more likely to take up a check than their male counterparts but that this increased likelihood attenuates with age.

There was also evidence that the direction of the association between level of risk and uptake varied according to the specific risk factor under investigation, with medical risk (e.g., family history) being associated with higher uptake and lifestyle risk (e.g., smoking status) being associated with lower uptake. This complexity is an important finding and warrants further investigation as patients at high risk of CVD are those who services want and need to engage with most urgently. These results mirror similar findings on engagement with healthcare services whereby those demonstrating high risk are less likely to attend health appointments [10, 37].

There is also a mixed picture in relation to ethnicity across the studies. For example, three studies reviewed here found that attendance was significantly higher in patients from South Asian, Asian and Black backgrounds [20, 24, 25], while another found that uptake was highest in Asian-Indian, Black-Caribbean and White British groups, but lowest in Black African groups [26]. In addition, others reported that uptake did not differ by patient ethnicity [9, 19]. Further complication arose from the finding that many of the studies showed high levels of missing ethnicity data [9, 20, 24–26], with Dalton and colleagues [24] finding that ethnicity data was missing for 31.8% of invited patients and 37.9% of NHSHC attendees. Whilst Artac and colleagues [20] specified that missing ethnicity data was sometimes due to patients being unwilling to disclose this information, Cook and colleagues [26] commented that high levels of missing data were due to GP practices failing to routinely update and audit their records, with Coghill and colleagues also commenting that poor recording of ethnicity by practices precluded the possibility of investigating the association between ethnicity and attendance in their study [22]. The complexity in uptake patterns revealed by this review highlights the importance of accurate and detailed ethnicity recording when investigating NHS Health Check uptake, as patients from specific ethnicities (e.g., Black African, Any Other White) can demonstrate different uptake patterns to the broader group level (e.g., Black, White).

The effect of deprivation on uptake of NHSHCs also varied across the studies and seemed to be influenced in some cases by whether or not analyses were adjusted for other predictor variables. Unfortunately, no studies investigated whether the impact of different invitation methods varied by deprivation levels. It is therefore not possible to assess which invitation strategies could be best used to engage those from the most deprived quintiles, who (as the majority of studies reviewed here found) were less likely to take up their NHSHC compared to patients within the least deprived quintiles. These findings dovetail with the findings of a large scale report that revealed that more affluent patients were more likely to respond to the invitation than less affluent patients (although overall coverage was higher among those from deprived communities [38]). This, alongside consistent evidence for variation between healthcare practices, demonstrates how important local context may be on the uptake of NHSHCs and indeed all health services. It is possible that the variation in uptake between individual practices may be a result of high correlations between individual practices and deprivation levels (e.g. [28],), a suggestion that is supported by the finding that adding GP practice to analyses impacted the relationship between deprivation and uptake in one study [19]. Indeed, other studies in this review (e.g. [21],) used the practice’s postcode as a measure of IMD for patient uptake, demonstrating how interlinked these factors are in research. However, the importance of considering other GP practice-specific factors when exploring reasons for uptake should not be ignored. This includes factors pertaining to the local delivery of the programme, such as the invitation process and whether opportunistic checks are conducted. Qualitative work in this area can also help to shed light on practice-specific factors that impact uptake, such as a lack of convenient appointment times and difficulty booking due to waiting lists [39, 40].

### Limitations of the review

One limitation is that in this instance, it was not possible to conduct a meta-analysis due to the substantial variation in the design of included studies [18]. An additional limitation is that this systematic review focused exclusively on literature on NHSHCs, possibly excluding relevant research from other programmes. However, this decision was made because, as far as the authors are aware, there are no other population-level preventative health check programmes with the same scope as the NHSHC, and there have been an increasing number of requests from LG for evidence on what works to increase uptake specifically for NHSHCs.

The search strategy identified many studies investigating interventions to increase uptake of NHSHCs, especially within hard to reach groups, however these were often conducted as service evaluations by local areas (e.g. [41],). Unfortunately, due to the designs of these studies, they could not be included in this systematic review, but they may hold important information about implementation locally. If local government were to collaborate with academics to utilise more robust research designs and facilitate more vigorous evaluations, this would enable more evidence to be collected more easily about programmes such as NHSHCs to help inform best practice. Increasingly, there are funding opportunities available for implementation research and academics keen to find local areas to test promising interventions. A recent strategy published by Public Health England’s Behavioural Insights team (PHEBI) makes steps towards this goal as it was developed with the aim of encouraging a greater integration of traditionally academic behavioural and social science disciplines into public health practice [42].

Finally, this review only included studies with quantitative measures of uptake and did not investigate qualitative work on the experiences of patients and practitioners in regards to NHSHC invitation processes, NHSHC appointments, and potential barriers and facilitators to uptake. A number of qualitative studies were identified throughout the search process and future reviews could synthesise these findings to gain a deeper insight into the factors that influence uptake from the perspective of patients and practitioners.

## Conclusions

This review found that, despite being the most widely used invitation method, letter invitations were less effective than telephone and face-to-face invitations (although one study revealed that this pattern may differ by patient gender and ethnicity). Nevertheless, there is evidence that letter invitations can be successfully enhanced using behavioural insights to improve uptake, which may be beneficial to services for whom telephone invitations are too costly. Our findings suggest that practices may need to consider additional targeted approaches to encourage groups who were found to have lower uptake rates of NHSHCs (namely younger cohorts, men, and those considered high risk according to lifestyle factors) to attend a NHSHC, however it was unfortunately not possible to find sufficient literature to identify which approaches may be best placed to reduced health inequalities. This review also finds that individual practice characteristics play a role in influencing uptake. As well as exploring how different demographic groups respond to invitation methods and interventions, further research is needed to understand the specific practice characteristics that impact NHSHC uptake, and whether the majority of such variation is due to the demographic characteristics of the patient list, or whether it is due to other factors such as availability of appointments.

With lifestyle factors now the biggest cause of death in the Western World it is imperative to reduce these lifestyle risk factors to achieve better health and wellbeing and reduce the associated health care costs.. It is clear further research is needed to help us identify effective ways of engaging people in preventative programmes such as the NHS HC. Understanding what interventions and invitation methods increase the uptake of NHSHCs, and identifying whether the efficacy of these interact with broader patient and contextual factors, will enable us to better support patients to reduce their risk. It is clear further research is needed to help us identify effective ways of engaging people in preventative programmes such as the NHS HC.

## Supplementary information

**Additional file 1.** Search Strategy.

**Additional file 2.** Database Hits.

**Additional file 3.** Data Extraction Table.

**Additional file 4.** List of Full Text Exclusions.

**Additional file 5.** Quality Assessment Tool.

## Data Availability

Data sharing is not applicable to this article as no datasets were generated or analysed during the current study. All relevant materials for replicating this research (e.g., search strategies) are provided, and the reviewed studies are clearly referenced so that other researchers can access and assess the conclusions drawn in this paper. The data extraction table is provided in the additional files, which identifies the results that were drawn from various papers to inform the conclusions of this review.

## Abbreviations

BMI: Body mass index
CVD: Cardiovascular disease
DARE: Database of Abstracts of Reviews of Effects
GP: General practitioner
LG: Local government
NHSHC: National Health Service Health Check
PICOS: Patient-Intervention-Comparator-Outcome-Study type
PRISMA: Preferred Reporting Items for Systematic reviews and Meta-Analyses
