# Supplementary material for: A systematic review of factors influencing NHS health check uptake: invitation methods, patient characteristics, and the impact of interventions
Source: BMC Public Health. 2020 Jan 21;20:93. doi: 10.1186/s12889-019-7889-4 (PMC6975079; doi:10.1186/s12889-019-7889-4)
Supplement: Supplementary file 1 — Additional file 1. Search Strategy. [file 12889_2019_7889_MOESM1_ESM.docx]

Appendix 1 – Search Strategy

Table 1 Search Strategy

| # | Search Term |
| --- | --- |
|  | “NHS Health Check” |
|  | NHS Health Check* |
|  | (nhs and health check*) |
|  | intervention* |
|  | invit* |
|  | opportuni* |
|  | appointment* |
|  | Communit* |
|  | offer* |
|  | Encourage* |
|  | “hard to reach” |
|  | inequalit* |
|  | depriv* |
|  | divers* |
|  | minorit* |
|  | Uptake |
|  | (take up or taking up) |
|  | accept* |
|  | attend* |
|  | book* |
|  | appointment* |
|  | S1 or S2 or S3 |
|  | S4 or S6 or S7 or S8 or S9 or S10 or S11 or S12 or S13 or S14 or S15 |
|  | S16 or S18 or S19 or S20 or S21 |
|  | S22 and S23 and S24 |

"NHS Health Check" OR nhs health check* OR (nhs and health check*)

AND

intervention* OR invit* OR opportunit* OR appointment* OR communit* OR offer* OR encourage* OR "hard to reach" OR inequalit*OR depriv* OR divers* OR minorit*

AND

uptake OR (take up OR taking up) OR accept* OR attend* OR book* OR appointment*

For the Cochrane Library the following terms were used;

NHS health Check OR health check OR check up

AND

“uptake” OR “attend”
