# Supplementary material for: A systematic review of factors influencing NHS health check uptake: invitation methods, patient characteristics, and the impact of interventions
Source: BMC Public Health. 2020 Jan 21;20:93. doi: 10.1186/s12889-019-7889-4 (PMC6975079; doi:10.1186/s12889-019-7889-4)
Supplement: Supplementary file 2 — Additional file 2. Database Hits. [file 12889_2019_7889_MOESM2_ESM.docx]

Appendix 2 – Hits by Database

Table 2 Hits by database (up to August 2016)

| Database | Number of References | Duplications within own database | Distinct References |
| --- | --- | --- | --- |
| EBSCO Host (Including CINAHL Plus with full text, Psych Info, Psych Articles and MEDLINE) | 404 | 165 | 239 |
| Ovid (including Embase) | 326 | 23 | 303 |
| SCOPUS | 160 | 4 | 156 |
| Web of Science | 152 | 4 | 148 |
| CDSR | 2 | 0 | 2 |
| Total | 1,044 | 232 | 848 |

Note: the Cochrane Controlled Register of Trials and Google Scholar was also searched manually, as noted in the Search Strategy section. However, no unique hits were identified through this process and so these databases are not included here.

Table 3 Hits by database (Jan 2016 - March 2018)

| Database | Number of References | Duplications (cumulatively) | Distinct References |
| --- | --- | --- | --- |
| Ovid Medline | 14 | 0 | 14 |
| Ovid Embase | 17 | 11 | 6 |
| Ovid PsycInfo | 2 | 2 | 0 |
| EBSCO CINAHL | 6 | 3 | 3 |
| CDSR and CTR | 57 | 5 | 52 |
| SCOPUS | 22 | 7 | 15 |
| Google Scholar | 14 | 1 | 13 |
| Total | 132 | 29 | 103 |

Note: One author (LP) subsequently identified an additional 6 references that had appeared in the 2016 searches and these were also removed, resulting in a final total of 97 unique search hits identified in the most recent search.
