# Supplementary material for: A systematic review of factors influencing NHS health check uptake: invitation methods, patient characteristics, and the impact of interventions
Source: BMC Public Health. 2020 Jan 21;20:93. doi: 10.1186/s12889-019-7889-4 (PMC6975079; doi:10.1186/s12889-019-7889-4)
Supplement: Supplementary file 3 — Additional file 3. Data Extraction Table. [file 12889_2019_7889_MOESM3_ESM.docx]

Appendix 3 – Study Data Extraction Table

| Author | Study Objective | Study Design | Participants | Method | Outcome Measure | Analysis Used | Results and Key Findings | Quality Score |
| --- | --- | --- | --- | --- | --- | --- | --- | --- |
| Artac et al., (2013) | To assess the  impact of a local financial  incentive scheme on uptake and statin prescribing in the first 2 years of the programme, including whether uptake and prescribing within the programme differed between socio- demographic groups. | Cross-sectional  study  Data extracted from GP system 2008 – 2011.  Sample size: Year 1 - 4,748  Year 2 - 35,364  patients invited to attend at NHS HC. | Year 1 - patients aged from  40 to 74 years with an estimated risk greater than  20% were targeted.  Year 2 - the remaining patients eligible for a Health Check.  Participant age range: 40- 74 years. | Invitation to attend based on level of risk  and practice incentivisation.  Individuals estimated to be at 20% or greater 10-year CVD risk were prioritized and invited for a Health Check in Year 1 of the programme (1 July 2008–30 November  2009).  In Year 2 (1 December 2009–31 March 2011), practices were incentivized to provide a Health Check to 30% of the remaining non-high risk population using opportunistic methods.  Existing risk factor data in electronic medical records was used to estimate CVD risk in all individuals aged from 40 to 74 years without diabetes and CVD, (hypertensive and chronic kidney disease patients were not excluded from the programme, as per national guidance, but were excluded from analyses) using the Joint British Societies 2 risk algorithm.  Context: GP practices in Hammersmith and Fulham. 27 out of 31 practices in year 1  and 29 practices in year 2. | Uptake of complete NHS Health Check | Multi-level logistic regression used to examine the predictors of uptake. | Year 1 – 1,551 (32.7%)  uptake  Year 2 – 7,076 (20.0%)  uptake  *Financial Incentives (Intervention)*   No stats reported in paper about effect of scheme    *Age*  55-64 age-group vs. 40-54 age-group (baseline) : AOR = 1.34, 95% CI = 1.11-1.61, p < .050  65-74 age-group vs. 40-54 age-group (baseline): AOR = 2.05, 95% CI = 1.67-2.52, p < .010    *Gender*   Year 1 (high-risk only) Female vs. male (baseline): AOR = 0.80, 95% CI = 0.67-0.94 p > .050  Year 2 (all eligible patients)  Female vs. male (baseline): AOR = 1.27, 95% CI = 1.20-1.35, p < .010    *Deprivation*  Year 1 analyses (high-risk patients only) IMD Tertile 3 vs. 1: AOR = 0.84, 95% CI = 0.69–1.01, p > .050 IMD Tertile 2 vs. 1: AOR = 0.94, 95% CI = 0.79-1.13, p > .050  Year 2 analyses (all eligible patients) IMD Tertile 3 vs. 1: AOR = 0.80, 95% CI = 0.73-0.87, p < .010  IMD Tertile 2 vs. 1: AOR = 0.84, 95% CI = 0.78-0.90, p < .010    *Ethnicity*  In Year 2, higher in patients of South Asian (AOR = 1.50, 95% CI = 1.25 – 1.78, p < .010) and Black ethnicity (AOR = 1.58, 95% CI = 1.43 – 1.75, p < .010) and Others (AOR = 1.16, 95% CI = 1.07 – 1.25), where White is baseline.    *Risk Factors*  Impact of comorbidities (medical risk) Year 1 AOR = 1.53, 95% CI = 1.31-1.80, p < .010 Year 2 AOR = 1.75, 95% CI = 1.64-1.87, p < .010  Impact of family history (medical risk) Year 1 AOR = 2.49, 95% CI = 2.15-2.90, p < .010 Year 2 AOR = 2.01, 95% CI = 1.87-2.16, p < .010  Impact of smoking status (lifestyle risk) Year 1 AOR = 0.71, 95% CI = 0.61-0.83, p < .010  Year 2 AOR = 0.83, 95% CI = 0.77-0.90, p < .010).  *Practice*  Practice List Size Year 2 (all eligible patients) >10,000 vs. <6000 (baseline): AOR = 6.05, 95% CI = 0.84-43.3, p < .010  Unexplained variance in models was interpreted as attributable to unmeasured practice factors Year 1 unexplained variance = 19.4%, 95% CI = 15.2-24.4%  Year 2 unexplained variance = 37.3%, 95% CI = 30.6-44.6% | 19 (strong) |
| Attwood et al., (2015) | To explore the  socio-  demographic characteristics of patients examining age, gender, ethnicity and deprivation level. | Cross sectional  study.  Data collected as part of a RCT pilot trial.  Sample size: 1,380 patients invited to attend a NHS HC. | Eligible patients were those  invited to attend a Health Check.  Participant age range: 40- 74 years. | Mailed invitation letters, mailed reminder  letters, face-to-face recruitment of eligible  patients attending pre-existing GP appointments and telephone recruitment.  Context: Four GP Practices in East of England | Participation  in the NHS  Health Check and in a nested physical activity trial. | Univariate  binary  logistic regression analyses were conducted to compare age, gender, ethnicity and IMD scores.  Interactions were explored using multivariate binary logistic regression analyses. | 1,165 (84%) uptake  *Age*  5% increase in likelihood of uptake with each additional year in age: OR = 1.05, 95% CI = 1.04-1.07, p < .010  *Gender*  Unadjusted model Female vs. male (baseline): OR = 1.50, 95% CI = 1.16-1.95, p < .050  Model adjusted for GP surgery effects Female vs. male (baseline): AOR = 1.29, 95% CI = 0.95-1.76, p > .050  *Deprivation*  Unadjusted analyses IMD Quintile 2 vs. 5: OR = 2.17, 95% CI = 1.39-3.38, p < .010 IMD Quintile 1 vs. 5: OR = 2.90, 95% CI = 1.84-4.58, p < .010  Adjusted analyses  IMD Quintile 2 vs. 5: AOR = 0.37, 95% CI = 0.18-0.67, p < .050  IMD Quintile 1 vs. 5: AOR = 0.42, 95% CI = 0.20 – 0.88, p < .050  *Ethnicity*  No difference between groups (OR = 0.59, 95% CI = 0.21-1.57)  *Practice*  Significant variance in uptake by practice X2 = 74.61, df = 2, p < .005  Adjusting analyses for GP Practice had a substantial effect on the strength and direction of associations between socio-demographic variables (specifically gender and IMD quintile; see relevant sections above) and uptake. | 20 (moderate) |
| Cochrane  et al (2012) | To evaluate the  influence of individual and  practice level factors on health check outcomes | Cross sectional  review  Data - Aug 2009  – Jan 2010 (First 6 months of programme.)  Sample size: 10,483 patients invited to attend an NHS HC. | Dedicated software was  used to stratify practice lists by estimated 10-year  CVD risk using the British Societies 2 risk score.  Patients free of established vascular disease and having  a CVD risk of ≥20% over the next 10 years were considered eligible for a HC.  Participant age range - 32 – 74 years (only 28 patients below 40 years) were  selected from general practice. | Each practice was provided with a list of  patients to invite. Practice nurses or project support workers in each practice  went through their lists systematically contacting patients until all eligible patients had been invited. Up to 3 reminder letters were sent.  Practices receive a per capita payment of  £15 - £20 as part of a LES (depending on  the level of service provided) for each patient who completes an NHS HC.  Context: 37 out of 57 GP Practices in Stoke  on Trent | Attendance at  health check and uptake of  treatment. | Difference  in proportions  – z tests. Multi-level non-linear regression analysis conducted to evaluate potential effects of the higher level  predictors  of practice size and the degree of deprivation on outcome measures. | 6,634 (63.3%) invited  responded, 4,580 (43.7%) attended a HC, 3,127  (29.8%) took up treatment.  *Age*  Overall effect of age-group: OR = 1.64, 95% CI = 1.51–1.77, p < .001  *Gender*  Female vs. male (baseline): OR = 0.70, 95% CI = 0.58-0.84, p < .001  *Deprivation*  Lowest attendance in tertile 3 (attendance rate = 42.6%, p < .050) Highest attendance in tertile 1 (attendance rate = 48.4%, p < .050  Deprivation was no longer significant when analyses were adjusted for gender, age, risk category and practice size  AOR = 1.12, 95% CI = 0.96 – 1.30  *Level of risk*  Risk category (combination of medical and lifestyle risk)  OR = 0.90, 95% CI = 0.80-1.02, p > .050 and <.100  *Practice*  Practice Size AOR = 1.03, 95% CI = 0.88-1.20, p > .100  Variance accounted for by individual practices = 12.7%, p < .001 | 19 (strong) |
| Coghill et al., 2018 | To assess the association between NHS Health Check uptake and socio-demographic characteristics such as age, sex, ethnicity and level of deprivation. | Cross-sectional observational study. Data was collected from June 2010 until October 2014. Thirty-eight practices in Bristol agreed to take part. A total of 31,881 patients from participating practices were invited for a Health Check. | Eligible patients were aged 40-74 years and had no pre-existing CVD, hypertension, ischaemic heart disease, stroke or ischaemic attacks, atrial fibrillation, heart failure, peripheral arterial disease, chronic kidney disease, familial hypercholesterolaemia, diabetes and current statin prescription. | Cross-sectional data obtained from electronic patient record databases. Age was divided into five groups, deprivation was measured according to IMD quintile, ethnicity was categorised in line with Good Clinical Practice Guidelines.  Context: 38 GP practices in Bristol. | Completion of a NHS Health Check. | Descriptive statistics summarised patient characteristics. Logistic regression was used to investigate associations between patient characteristic and attendance at NHS Health Check. | *Age*  Overall effect of age p < .001  50-59 years vs. ≤ 49 years (baseline): AOR = 1.36, 95% CI = 1.21-1.53  60-69 years vs. ≤ 49 years (baseline): AOR = 2.19, 95% CI = 1.80-2.68  70+ years vs. ≤ 49 years (baseline): AOR = 2.53, 95% CI = 1.89-3.39)  *Gender*  Male vs. female (baseline): AOR = 0.73, 95% CI = 0.67-0.80, p < .001  *Deprivation*  Non-significant effect of deprivation on uptake (p = .053) | 20 (moderate) |
| Cook et al., 2016 | To identify  systematic differences  among socio- demographic differences in the uptake of  NHS Health Checks in a culturally  diverse town of  England accounting for age, gender, deprivation and examine the methods of invitation to determine if some methods are more  successful than  others for specific population groups by ethnicity and gender. | Cross sectional  study  Data set from April 2013 –  March 2014 analysed.  Sample size: 13,063 patients offered an NHS HC. | All patients who were  eligible for an NHS Health Check in Luton over the 12  month period 1^st^ April 2013  – 31^st^ March 2014.  This included patients who were eligible, had been offered and who had an NHS Health Check.  Excluded anyone who is receiving treatment or support for coronary heart disease, chronic kidney disease (CKD) which has been classified as stage 3, 4 or 5 within, diabetes, hypertension, atrial fibrillation, transient ischaemic attack, hypercholesterolemia,  heart failure, peripheral  arterial disease, stroke are not eligible. Also anyone who has been prescribed statins, and during a previous NHS Health was found to have a 20 % or higher ten year risk of developing CVD.  Participant age range: 40 – 74 years. | Investigating the impact of ethnicity and  gender and method of invitation on uptake of NHS Health Check.  Invitation method was categorised by 1) verbal (face to face) invitation (invited at GP Practice) 2) contacted by telephone by GP practice 3) written (invitation letter sent from GP practice to attend NHS HC)  Context: All 30 GP Practices in Luton | Uptake of  NHS Health Check | Chi Square  analysis to determine  the actual usage of NHS HCs for each ethnic  group. | 5,703 (44%) uptake.  Note:  UR = Uptake rate  *Invitation Method*  Highest uptake rates after letter invitation for Mixed White and Asian male (UR = 0.70, 95% CI = 0.40 – 0.92, p < .010), Chinese female (UR = 0.62, 95% CI = 0.35 – 0.84, p < .010); least effective for any other white males (male UR = 0.19, 95% CI = 0.27 – 0.22, p < .001), African males (UR = 0.23, 95% CI = 0.19-0.28), p < .010), Mixed: Other females (UR = 0.05, 95% CI = 0.00 – 0.20, p < .050), Pakistani female (UR = 0.21, 95% CI = 0.15 – 0.28, p < .050), any other white females (UR = 0.22, 95% CI = 0.19 – 0.25, p < .001) and African females (UR = .023, 95% CI = 0.19 – 0.28, p < .050).  Face-to-face most effective for White British male (UR = 0.72, 95% CI = 0.70 – 0.82, p < .001), White Irish female (UR = 0.93, 95% CI = 0.74 – 0.99, p < .050) and White British female (UR = 0.79, 95% CI = 0.72 – 0.85, p < .001), least effective for Bangladeshi male (UR = 0.43, 95% CI = 0.23 – 0.64, p < .001), Pakistani male (UR = 0.47, 95% CI = 0.24 – 0.71, p < .050).  Telephone most effective Pakistani males (UR = 1.00, 95% CI = 0.38 – 1.00, p < .010), Pakistani females (UR = 1.00, 95% CI = 0.83 – 1.00, p < .001), White/Black Caribbean females (UR = 1.00, 95% CI = 0.83 – 1.00, p < .001), White Irish females (UR = 0.96, 95% CI = 0.84 – 0.99, p < .001) and Asian Other females (UR = 0.76, 0.53 – 0.91, p < .001), but least effective for any other white males (UR = 0.10, 95% CI = 0.04 – 0.21, p < .001), White British female (UR = 0.00, 95% CI = 0.00 – 0.01, p < .001) and White Other females (UR = 0.08, 95% CI = 0.01 – 0.22, p < .001).  *Age*  Highest uptake in 65-69 (male uptake = 71%, p < .001, female uptake = 62%, p < .001) and 70-74 age-groups (male uptake = 68%, p < .001, female uptake = 80%, p < .001)  *Gender*  Female uptake rate = 50%, p < .001; male uptake rate = 38%, p < .001  *Deprivation*  Lowest uptake in Quintile 1 with uptake rates of 0.31 and 0.38 for males and females respectively, p < .001  Highest uptake in the Quintile 5, with uptake rates of 0.53 and 0.60 respectively, p < .001  *Ethnicity*  Higher uptake amongst Mixed White and Asian males (UR = 0.91, 95% CI = 0.66 – 0.99, p < .001), Caribbean males (UR = 0.69, 95% CI = 0.62 – 0.76, p < .001),Chinese males (UR = 0.67, 95% CI = 0.45 – 0.84, p < .010), Chinese females (UR = 0.93, 95% CI = 0.72 – 0.99, p < .001), White/Black Caribbean females (UR = 0.77, 95% CI = 0.63 – 0.87, p < .001), White Irish females (UR = 0.72, 95% CI = 0.64 – 0.80, p < .001), and Black Caribbean females (UR = 0.71, 95% CI = 0.64 – 0.77, p < .001).  Lower uptake amongst any other white patients (male UR = 0.27, 95% CI = 0.24 – 0.30, p < .001; female UR = 0.35, 95% CI = 0.31 – 0.38, p < .001) and Black African females (UR = 0.42, 95% CI = 0.37 – 0.47, p < .010). | 23 (moderate) |
| Dalton et  al (2011) | To examine  uptake of the Health Checks programme in the first year of implementation and explore whether participation in the programme differed with patient and practice characteristics. | A cross  sectional study  Data - September 2008  – January 2010.  Sample size: 5,294 patients invited to attend a HC. | Disease free individuals  estimated to be at or greater than a 20% 10-year risk of a CVD event were targeted in the first year of the programme (1^st^ Sept 2008 – 31 Aug 2009). It  included patients with  diagnosed hypertension and those prescribed statins.  Participant age range -35 – 74 years. | The PCT provided each general practice  with a list of patients to be invited in year one, and the practice then contacted patients by an invitation letter inviting them to attend a Health Check.  Local Enhanced Service scheme whereby practices receive 15 per person screened. Disease-free individuals estimated to be at, or greater than, a 20% 10-year risk of a CVD event were targeted in the first year of the programme (1 September 2008 to 31 August 2009); the method of risk estimation is detailed subsequently.  Context: 29 of 86 GP practices in Ealing, North London. | Attendance  for screening | Multi-level  logistic regression to analyse health check attendance. | 2,370 (44.8% uptake).  *Age & Gender*  55-64 age-group vs. 35-54 age-group (baseline) : AOR = 1.74, 95% CI = 1.34-2.25, p < .001  65-74 age-group vs. 35-54 age-group (baseline) : AOR = 2.27, 95% CI = 1.47-3.50, p < .001  Significant age x gender interaction; women in the youngest age-group (35-54 years) more likely to attend than men in the same age category: AOR = 1.71, 95% CI = 1.03-2.85, p = .037  *Deprivation*  No significant effect of deprivation (p>.050)  *Ethnicity*  When compared to White British patients, uptake higher in patients of South Asian (AOR = 1.71, 95% CI = 1.29 – 2.27, p < .001) and mixed ethnic backgrounds (AOR = 2.42, 95% CI = 1.50 – 3.89, p = .015).  *Risk factor*  Smoking status (lifestyle risk)  Yes vs. no (baseline): AOR = 0.88, 95% CI = 0.75-1.92, p = .097  *Practice*  Practice List Size  <3000 vs. 3000-5999 (baseline): AOR = 2.53, 95% CI = 1.09-5.84, p = .030 ≥6000 vs. 3000-5999 (baseline): AOR = 0.79, 95% CI = 0.33-1.88, p = .599  Variance in models accounted for by practice = 28% (VPC = 0.28) | 19  (striong) |
| Gidlow et  al (2014) | To assess the  impact of  invitation method and geographical proximity on uptake in deprived urban communities. | Observational  Cohort Study  Data – September 2010  – February 2014.  Sample size: 4,855 patients invited for an NHS Health Check. | Patients who had been  invited for a Health Check  between Sept 2010 and Feb 2014.  Participant age range - 40 – 74 years. | Method of invitation method – verbal/  telephone invite either alone or in combination with an invitation letter.  Context: Five out of 53 GP Practices is Stoke on Trent | Attendance at  NHS Health Check | Two-stage  binary  logistic regression analysis was used to explore predictors of Health Check attendance. | 2,989 (61.6% uptake).  *Invitation Method*  Using telephone/face-to-face alone or with letter = higher attendance (OR 2.87, 95% CI = 2.26 – 3.64, p < .001)  *Age*  4% increase in likelihood of uptake with each additional year of age : OR = 1.04, 95% CI = 1.03-1.04, p < .001  *Gender*  Female vs. male (baseline): OR = 1.47, 95% CI = 1.30-1.68, p < .001  *Deprivation*  IMD Quintile 5 vs. 1: OR = 1.59, 95% CI = 1.23-2.05, p < .001  IMD Quintile 4 vs. 1: OR = 1.30, 95% CI = 1.06 – 1.61, p = .014  IMD Quintile 3 vs. 1: OR = 1.24, 95% CI = 1.03 – 1.49, p = .022 IMD Quintile 2 vs. 1: OR = 1.11, 95% CI = 0.87-1.43, p = .395  Overall effect of deprivation p = .008  *Ethnicity*  No difference by group (X^2^ = 0.769, p = .380)  *Practice*  Variation in uptake by practice X2 = 336.9, p<.001  Variation in uptake by distance to practice  X^2^ = 0.478, p = .924 | 19 (strong) |
| McDermott et al., (2018) | To investigate the effect of sending a Question-Behaviour Effect questionnaire (with or without a financial incentive for returning the questionnaire) ahead of standard invitation letters on uptake of NHS Health Checks | Three-arm randomised control trial across two London boroughs.  Sample size: 12,681 participant | All participants eligible for a NHS Health Check at 18 practices across two London boroughs were included.  Participants’ ages ranged from 40-74. | The standard letter invitation method (Control) group was compared against two groups who received a Question-Behaviour Effect questionnaire in the post 7 days before the arrival of the standard letter. One of these groups received a financial incentive for returning the questionnaire (£5 retail voucher). The questionnaire aimed to encourage Health Check uptake by asking participants about their beliefs concerning attending Health Checks.  All participants were sent a standard reminder letter at 3 months if they had not responded to the original invitation. | Completion of a health check within 6 months of invitation. | General estimating equations (GEE) – binomial marginal model with identity link. Subgroup analyses explored demographic characteristic influence.  Significance value set at p<.0167 to account for multiple comparisons.  Complier-Average Causal Effect analysis was performed for per-protocol analysis. | *Invitation Intervention*  No significant effect of questionnaire (p = .070) or questionnaire plus incentive (p = .054)  Complier-Average Causal Effect analysis showed significant increase of 6% in the questionnaire group compared to standard letter (95% CI = 0.8 – 11.3%, p = .024) and 5.9% increase in the questionnaire plus incentive (95% CI = 0.8 – 10.9%, p = .022).  More likely to return questionnaire if female, older, lower deprivation (no statistics reported for this).  *Age*  60-74 age group vs. 40-59 age-group (baseline): OR = 1.43, 95% CI = 1.20-1.71, p < .001  *Gender*  Male vs. female (baseline): AOR = 0.74, 95% CI = 0.69-0.80, p < .001  *Deprivation*  IMD Quintile 4 vs. 1: AOR = 2.78, 95% CI = 1.87-4.12, p < .001 IMD Quintile 3 vs. 1: AOR = 1.15, 95% CI = 0.95-1.39, p = .156 IMD Quintile 2 vs. 1: AOR = 1.09, 95% CI = 0.95-1.24, p = .214 (Note, no data was collected from Quintile 5 in this study)  *Ethnicity*  Higher uptake amongst Asian (OR = 2.03, 95% CI = 1.63 – 2.67, p < .001), African/Caribbean (OR = 2.15, 95% CI = 1.86 – 2.49, p < .001) and mixed (OR = 3.09, 95% CI = 2.07 – 4.62, p < .001) ethnicity groups compared to White patients. | 16 (strong) |
| Sallis et al., (2016) | This study  aimed to test the impact of an enhanced invitation letter on attendance at an NHS HC appointment compared to the standard national  template letter. | Quasi  Randomised Controlled Trial  Sample size: 3,511 patients invited to attend an NHS HC. | All patients eligible for an  NHS HC in 2013/14  registered at one of the four practices in Medway  were included.  Participant age range - 40- 74 years. | Comparing the standard national invitation  template letter (control) to an enhanced invitation letter using insights from behavioural science (intervention). The intervention letter includes i) simplification reducing letter content for less effortful processing ii) behavioural instruction - action focused language iii) personal salience - appointment due rather than invited and iv) addressing implementation intentions with a tear off slip to record the  date, time and location of the appointment**.**  Invitation letters were sent by post May 2013.  Context: Four GP practices in Medway, Kent, were purposively selected due to having large numbers of eligible patients, suitable IT systems and centrally administered systems for distributing the letters. | Attendance at  NHS Health Check | Logistic  Regression explored the association between control and intervention group and attendance  at a health  check. | 1,102 (31.4%) uptake.  29.3% - control letter 33.5% - Intervention letter.  *Invitation Intervention*  Invitation increased uptake (AOR = 1.26, 95% CI = 1.09 – 1.47, p < .010).  Interaction between letter and practice (p < .001) comparing practices 1 and 4 (AOR = 1.76, 95% CI = 1.18 – 2.64).  *Age*  62% increase in likelihood of uptake with every additional 10 years in age: AOR = 1.62, 95% CI = 1.50-1.75, p < .010  *Gender*  Female vs. male (baseline): AOR = 1.50, 95% CI = 1.29-1.74, p < .010)  *Deprivation*  IMD Quintile 5 vs. 1: AOR = 1.61, 95% CI = 1.14-2.26, p < .010  All other comparisons against Quintile 1 (baseline) p > .010  *Practice*  Of the five practices studied, one (used as baseline in analyses) had significantly higher uptake rates than all others (all p < .010). | 16 (strong) |
