# Supplementary material for: A systematic review of factors influencing NHS health check uptake: invitation methods, patient characteristics, and the impact of interventions
Source: BMC Public Health. 2020 Jan 21;20:93. doi: 10.1186/s12889-019-7889-4 (PMC6975079; doi:10.1186/s12889-019-7889-4)
Supplement: Supplementary file 5 — Additional file 5. Quality Assessment Tool. [file 12889_2019_7889_MOESM5_ESM.docx]

**Appendix 4 – Quality Assessment**

To accommodate and appropriately assess studies with different designs, an adapted version of the quality assessment tool for quantitative studies developed by the Effective Public Health Practice Project (EPHPP) was developed, incorporating questions from the US National Institutes of Health National Heart, Lung, and Blood Institute (NHLBI, 2014) for observational cohort and cross sectional studies and the Downs and Black (1998) checklist for randomised and non-randomised studies to create an appropriate quality assessment checklist.

The quality assessment included 16 questions considering the appropriateness of study design to the research objective, the risk of bias, choice of outcome measure, quality of sample size (power) and analysis, quality of reporting, quality of the intervention and its generalisability. Each question was scored with a final rating indicating the strength of the quality of the paper. The reviewers scored the papers independently. For the 2016 searches, an independent interrater reliability score was calculated using Cohen’s kappa (Cohen, 1960), showing substantial agreement between the raters (*k* = 0.772, *p* < .0005) (McHugh, 2012). The two raters then met to resolve any discrepancies. When a final assessment of a component was unclear the lower assessment was set, adopting a conservative decision. For the 2018 searches, perfect agreement was achieved for the quality assessment of the two papers added to the review.

The minimum score is 16 representing the strongest study quality and the maximum score is 32 representing the weakest study quality.

When a final assessment of a component was unclear the lower assessment was set, adopting a conservative decision.

| Score | Rating |
| --- | --- |
| 16- 19 | Strong |
| 20 – 23 | Moderate |
| 24 – 32 | Weak |

Table 4 Quality Assessment of included papers.

|  | Artac, Dalton, Majeed et al (2013)  Uptake of the NHS Health Check programme in an urban setting | Attwood, Morton & Sutton (2015)  Exploring equity in uptake of the NHS Health Check | Cochrane, Gidlow, Kumar et al (2012)  Cross-sectional review of the response and treatment uptake from the NHS Health Checks programme in Stoke on Trent | Coghill et al., (2018) | Cook et al (2016)  Who use NHS Health Checks? Investigating the impact of ethnicity and gender and method of invitation on uptake of NHS health checks | Dalton, Bottle, Okoro et al (2011)  Uptake of the NHS Health Checks programme in a deprived, culturally diverse setting | Gidlow, Ellis, Randall et al (2014)  Method of invitation and geographical proximity as predictors of NHS Health Check uptake | Sallis et al (2016)  The effectiveness of an enhanced invitation letter on uptake of NHS Health Checks in Primary Care | McDermott et al (2018)  Enhanced invitations using the question-behavior effect and financial incentives to promote health check uptake in primary care |
| --- | --- | --- | --- | --- | --- | --- | --- | --- | --- |
| 1. Was the research question or objective clearly stated?   Yes – 1  No – 2 | 1 | 1 | 1 | 1 | 1 | 1 | 1 | 1 | 1 |
| 1. Are the individuals selected to participate in the study likely to be representative of the target population?   Yes – 1  No – 2  Unable to determine – 2 | 1 | 1 | 1 | 1 | 1 | 1 | 1 | 1 | 1 |
| 1. Were inclusion and exclusion criteria pre-specified and applied uniformly to all participants?   Yes - 1  No - 2  Unable to determine – 2 | 1 | 1 | 1 | 1 | 1 | 1 | 1 | 1 | 1 |
| 1. Are the characteristics of the participants included in the study clearly described?   Yes - 1  No – 2 | 1 | 1 | 1 | 1 | 1 | 1 | 1 | 1 | 1 |
| 1. Are the outcome measures used appropriate/ relevant to the research question?   Yes - 1  No – 2 | 1 | 1 | 1 | 1 | 1 | 1 | 1 | 1 | 1 |
| 1. Was a sample size justification, power description, or variance and effect estimates provided?   Yes - 1  No – 2 | 1 | 1 | 1 | 2 | 2 | 1 | 1 | 1 | 1 |
| 1. Were participants randomised to intervention/ control groups?   Yes – 1  No – 2  Unable to determine – 2 | 2 | 2 | 2 | 2 | 2 | 2 | 2 | 1 | 1 |
| 1. Was the method of randomisation described appropriate?   Yes – 1  No – 2  Not applicable - 2  Unable to determine – 2 | 2 N/A | 2 N/A | 2 N/A | 2 | 2 | 2 N/A | 2 N/A | 1 | 1 |
| 1. Are the interventions of interest clearly described?   Yes – 1  No – 2 | 1 | 2 | 1 | 2 | 2 | 1 | 1 | 1 | 1 |
| 1. Were the outcome assessors blinded to the exposure status of participants?   Yes -1  No - 2  N/A – 1  Unable to determine – 2 | 2 U | 2 | 2 | 1 | 2 | 2 U | 2 | 1 | 1 |
| 1. Were the study participants unaware of the research question?   Yes -1  No - 2  Unable to determine – 2 | 1 | 1 | 1 | 1 | 1 | 1 | 1 | 1 | 1 |
| 1. Was the study response rate reported?   Yes -1  No – 2  Unable to determine – 2 | 1 | 1 | 1 | 1 | 1 | 1 | 1 | 1 | 1 |
| 1. Were lost to follow up rates reported?   Yes – 1  No – 2  N/A* - 1 | 1 N/A | 1 | 1 | 1 | 1 N/A | 1 N/A | 1 N/A | 1 N/A | 1 |
| 1. Are the outcome measures reported?   Yes - 1  No – 2 | 1 | 1 | 1 | 1 | 1 | 1 | 1 | 1 | 1 |
| 1. Are the statistical tests used to assess the main outcomes appropriate?   Yes – 1  No – 2  Unable to determine – 2 | 1 | 1 | 1 | 1 | 2 U | 1 | 1 | 1 | 1 |
| 1. Was there adequate adjustment for confounding factors in the analyses?   Yes -1  No – 2  Unable to determine – 2 | 1 | 1 | 1 | 1 | 2 | 1 | 1 | 1 | 1 |
| Total | 19 | 20 | 19 | 20 | 23 | 19 | 19 | 16 | 16 |

*Withdrawals and drop outs do not occur when the total local or national level data collection is done. N/A – Not applicable to this study design.
